# Two-Phase Biocatalysis in Microfluidic Droplets

**DOI:** 10.3390/bios11110407

**Published:** 2021-10-21

**Authors:** Lanting Xiang, Felix Kaspar, Anett Schallmey, Iordania Constantinou

**Affiliations:** 1 Institute for Microtechnology, Technische Universität Braunschweig, 38124 Braunschweig, Germany; l.xiang@tu-braunschweig.de; 2 Zentrum für Pharmaverfahrenstechnik (PVZ), Technische Universität Braunschweig, 38106 Braunschweig, Germany; a.schallmey@tu-braunschweig.de; 3 Institute for Biochemistry, Biotechnology and Bioinformatics, Technische Universität Braunschweig, 38106 Braunschweig, Germany; felix.kaspar@tu-braunschweig.de; 4 Chair of Bioprocess Engineering, Institute of Biotechnology, Faculty III Process Sciences, Technische Universität Berlin, 13355 Berlin, Germany; 5 Braunschweig Integrated Center of Systems Biology (BRICS), Technische Universität Braunschweig, 38106 Braunschweig, Germany

**Keywords:** microfluidics, two-phase biocatalysis, microfluidic droplets, enzyme

## Abstract

This Perspective discusses the literature related to two-phase biocatalysis in microfluidic droplets. Enzymes used as catalysts in biocatalysis are generally less stable in organic media than in their native aqueous environments; however, chemical and pharmaceutical compounds are often insoluble in water. The use of aqueous/organic two-phase media provides a solution to this problem and has therefore become standard practice for multiple biotransformations. In batch, two-phase biocatalysis is limited by mass transport, a limitation that can be overcome with the use of microfluidic systems. Although, two-phase biocatalysis in laminar flow systems has been extensively studied, microfluidic droplets have been primarily used for enzyme screening. In this Perspective, we summarize the limited published work on two-phase biocatalysis in microfluidic droplets and discuss the limitations, challenges, and future perspectives of this technology.

## 1. Introduction

Biocatalysis is an empowering technology in synthetic organic chemistry. The use of enzymes for chemical transformations often grants unparalleled chemo-, regio- and stereoselectivity, and even enables transformations that would be impossible to achieve with conventional chemical methods [1,2,3,4]. Owing to these advantages, recent years have witnessed a growing popularity of strategic enzymatic steps in the preparation of pharmaceuticals, natural products, and other fine chemicals [5,6,7,8,9,10]. By reducing the length of synthetic routes and the demand for laborious purification processes, biocatalytic transformations typically exhibit more favorable green chemistry metrics than their corresponding chemical counterparts [11,12]. As such, biocatalysis continues to evolve into the intuitive choice for the development of environmentally friendly chemical processes, both in academic as well as industrial settings [13]. However, the efficient application of enzymes is often hampered by the low water solubility of desired starting materials. As many compounds of interest in organic chemistry exhibit only limited hydrophilicity, substrate loadings in biocatalytic transformations are often restricted to the low millimolar range, far below the desired titers for industrial settings [11]. To increase the substrate concentrations and thereby decrease solvent use, several strategies and process designs have been established. Among these, the use of two immiscible liquid phases, an aqueous and a non-aqueous phase, the latter usually comprising an organic solvent [14,15,16] or ionic liquid [17,18], has emerged as an elegant and remarkably versatile approach. The enzymatic reaction typically takes place in the aqueous phase under substrate concentrations near the solubility limit, while the non-aqueous (commonly organic) phase acts as a substrate reservoir, continuously delivering the substrate to the aqueous phase. At the same time, the non-aqueous phase serves as a product sink, removing the reaction product from the aqueous phase. Beyond enabling exceptionally high net substrate concentrations, two-phase biocatalysis has several key advantages, including the prevention of inhibition effects or shift of reaction equilibria via continuous product removal, as well as straightforward product isolation via phase separation [19,20]. Despite these advantages, biphasic processes are yet to become a true standard technique in biocatalysis, as the design of efficient biphasic enzymatic transformations is challenging and requires non-trivial engineering. Another major challenge is the mixing of the two liquid phases which maximizes mass transfer but can also compromise the stability and hence the activity of the enzyme catalyst [21]. Moreover, stable emulsions can form when performing liquid–liquid two-phase reactions in batch under vigorous stirring, complicating phase separation and thus product isolation during subsequent down-stream processing [22]. The aforementioned challenges can be overcome with the use of microfluidic systems.

## 2. Biocatalysis at the Microscale

Flow chemistry has been performed in organic chemistry laboratories in academia and industry for the development, optimization, and intensification of chemical processes for several decades [23]. More recently, the first biocatalytic reactions performed in microfluidics were reported [24,25]; however, flow biocatalysis today remains in its infancy compared to flow chemistry. This is surprising considering the many advantages microfluidics offer, such as the possibility of continuously operated reactions, much higher surface-to-volume ratios and hence short diffusion paths, and tremendously increased mass and heat transfer rates [26,27]. These advantages are especially promising for liquid–liquid two-phase biocatalytic reactions that are often limited by mass transfer in batch [28]. As different physical effects influence fluid flow at the microscale compared to larger scales [29], fluid flow regimes unachievable on the macroscale (such as laminar flow) can be achieved in microchannels. Moreover, process development significantly benefits from microscale technology due to reduced consumption of reagents and time, speeding up process development and reducing development costs [26]. In the biocatalysis field, this has led to the development of microfluidics-based screening methods for activity-based screening of metagenomic libraries [30,31] or mutant libraries derived from enzyme evolution campaigns [32,33]. Additionally, enzyme characterization and process parameter estimation and optimization are also facilitated by the use of microfluidic devices (often microfluidic droplets) due to ultra-high throughput and low reagent consumption [23,34,35,36]. In addition to high-throughput screening and characterization possibilities, miniaturization provides a tool that allows researchers to gain an understanding of fundamental process principles and reaction characteristics. This is made possible by the fact that experimental conditions can be precisely and independently controlled in microfluidic systems. Although some processes optimized at the microscale cannot be directly scaled up, fundamental process understanding gained at the microscale can still facilitate process optimization in the macroscale. Finally, smaller volumes and better spatial and temporal reaction control in microsystems increase safety in biocatalytic reactions that involve, e.g., toxic reagents [37].

## 3. Droplet Generation in Microfluidic Systems

For two-phase biocatalysis in microfluidics, the two liquid phases need to be in contact to ensure fast mass transport across the interface. With the absence of turbulence at the microscale, molecular diffusion becomes the dominant mechanism of mass transfer. A liquid–liquid, two-phase flow in microchannels can be achieved through laminar flow or using microfluidic droplets.

**Figure 1:**
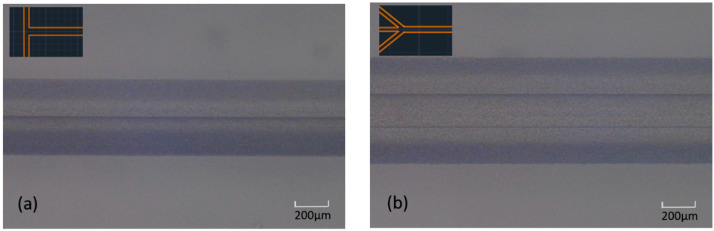
Laminar flow in a microfluidic channel: (**a**) two liquid phases flowing next to each other and forming two layers and one interface; (**b**) two liquid phases flowing next to each other and forming three layers and two interfaces. The microfluidic device architectures used to achieve these flow patterns are shown as inserts.

Laminar flow is dominant when Reynold numbers are low and is characterized by fluid streamlines parallel to the channel walls. The two phases flow next to each other in the form of two layers forming one interface (Figure 1a) or three layers forming two interfaces (Figure 1b). Two-layer flow profiles are commonly produced using T-junctions and three-layer laminar flow profiles using Ψ-junctions. The width of each phase, i.e., the volume each phase occupies in the channel, can be adjusted by changing the flow rate ratio between the two liquids. Such microsystems are often used for two-phase biocatalysis using immiscible hydrophobic substrates, as they provide advantages like short diffusion paths across the aqueous/non-aqueous interface and easy phase separation at the outlets [38,39].

**Figure 2:**
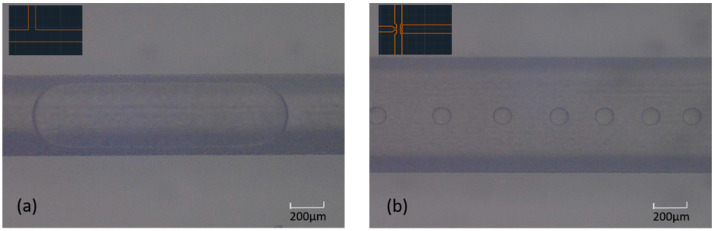
(**a**) Ellipsoidal microdroplet in a microfluidic channel/slug flow. (**b**) Spherical microdroplets in a microfluidic channel/droplet flow. The microfluidic device architectures used to achieve these flow patterns are shown in the inserts and discussed in detail below.

Similar to laminar flows, microfluidic droplets have also been employed as a tool for biphasic enzymatic transformations, however, to a much lesser extent. Microfluidic droplet formation requires two immiscible fluids (gas–liquid or liquid–liquid), referred to as the continuous phase (medium in which droplets flow) and the dispersed phase (droplets). In the context of two-phase biocatalysis, liquid–liquid droplets are formed using an organic and an aqueous phase. Each microdroplet can be regarded as an independent microreactor, with fast mass transfer taking place across the droplet boundary. In addition to efficient mass transfer, reactions in microfluidic droplets can greatly reduce the consumption of expensive reagents due to their tiny volumes (commonly pL to nL) and at the same time limit cross-contamination. There are two common droplet geometries; ellipsoidal (Figure 2a) and spherical (Figure 2b). Ellipsoidal droplets can be produced using T-, Y- and Ψ-junctions. They are larger compared to spherical microdroplets and their width is typically determined by the width of the microfluidic channel. The flow of large ellipsoidal droplets (slugs) in a microchannel is commonly referred to as slug flow. Spherical microdroplets are produced using flow focusing or co-flowing microfluidic structures and often have diameters much smaller than the width of the microchannel. Device geometries and droplet generation methods are discussed in detail below.

### 3.1. Microfluidic Droplet Production and Their Applications in Two-Phase Biocatalysis

Various applications of microfluidic droplets employed in the field of biocatalysis have been reported in the literature. These can be divided into three main categories: (1) microfluidic droplets for ultrahigh-throughput library screening, (2) microfluidic droplets for enzyme characterization and parameter estimation, and (3) intensification of two-phase biocatalytic reactions using microfluidic droplets or droplet-like fluid flows. The first two categories have already been reviewed comprehensively in recent literature and will not be further discussed in this perspective [23,30,33,34]. Herein, we focus on synthetically useful biocatalytic reactions employing two liquid phases, in an effort to highlight the benefits and limitations associated with droplet microfluidics when used to perform these reactions. Before we discuss specific examples of two-phase biocatalysis in droplets, we offer a relevant introduction into the production technology of droplet microfluidics.

### 3.2. Production Technology of Droplet Microfluidics

Droplet formation methods can be classified as passive or active. Passive methods primarily use microchannel architecture and flow rates to control the formation of droplets, while active methods employ external force fields (e.g., light, electricity). In droplet-based biocatalysis, active droplet formation methods are generally avoided as the used external force fields could harm the enzymes or cells used to facilitate the reactions. The most common passive hydrodynamic methods used to produce microdroplets can be categorized into three groups according to the microchannel architecture: T-junction, flow-focusing, and co-flowing devices. The geometry of the intersection where the two flows meet, the flow rate of each phase and the properties of the fluid (surface tension, viscosity, etc.) determine the local stresses that deform the liquid interface and cause droplet formation.

**Figure 3:**
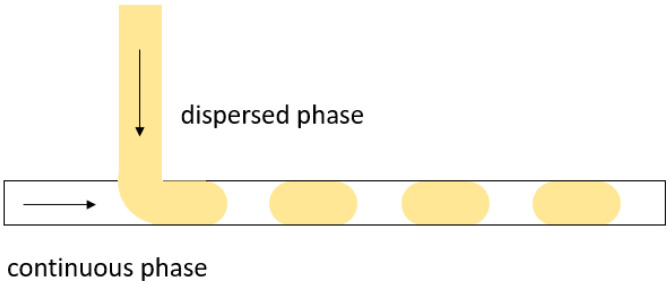
Droplet generation in a T-junction. Large droplets are formed in the channel, resulting in slug flow.

**T-junction**: T-junctions were first proposed by Thorsen et al. in 2001 [40]. Droplets in this device architecture are formed when two mutually immiscible fluids meet at the T-shaped microchannel intersection. Under the action of pressure and shear, the continuous phase cuts off the dispersed phase to form droplets. As seen in Figure 3, T-junctions have a simple architecture and are easy to fabricate, which makes them a popular choice for applications in biocatalysis. However, the droplet width is determined by the width of the microfluidic channel, and therefore, spherical droplets with smaller diameters cannot be produced. This can be a limiting factor when using these devices for two-phase biocatalysis, as the available droplet surface area can be quite small compared to that of smaller droplets that float in the middle of the channel. This can partly inhibit mass transfer across the droplet interface (e.g., diffusion of substrate from the organic to the aqueous phase), a critical step during biocatalysis.

T-junctions, and similarly Y-junctions, have been the most commonly used microfluidic architectures to perform biphasic biocatalysis experiments. Some examples include work from Pohar et al., who used microfluidic devices of this architecture for the synthesis of isoamyl acetate [41], and Tušek et al., in which Y-junction microfluidic devices were used to study hexanol oxidation [42]. In the work of Tušek, two-phase biocatalysis was performed in a droplet microreactor and a cuvette under the same conditions. A kinetic model and a reactor model were established based on the results of the biotransformation. Comparing the kinetic parameters estimated by the two measurements, the maximum reaction rate in the microreactor was estimated to be approximately 33 times higher than in the cuvette. Additionally, in the experiments performed in microfluidic droplets, almost no reaction inhibition or reverse reaction were observed.

**Figure 4:**
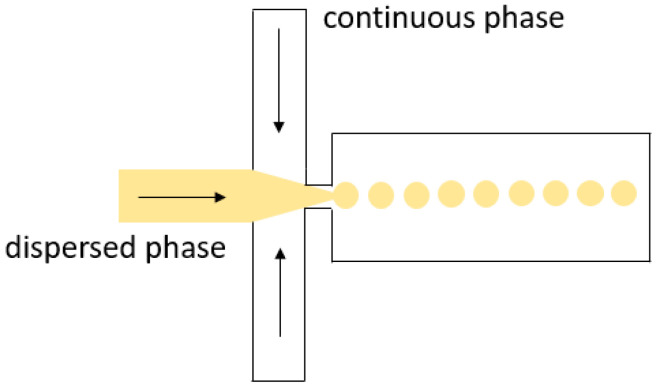
Droplet generation in a flow-focusing device. Small spherical droplets are formed in the channel resulting in droplet flow.

**Flow focusing**: The first micromachined flow-focusing system was introduced in 2001 by Ganan-Calvo et al. [43]. In 2003, Anna et al. used the flow-focusing geometry to form microfluidic water droplets in oil (water-in-oil droplets) and oil droplets in water (oil-in-water droplets) [44]. To generate microfluidic droplets, the continuous phase is injected into the microfluidic device through two side channels and is used to squeeze the dispersed phase, flowing through the central channel (Figure 4). The formation of microfluidic droplets is also facilitated by geometric constraints, such as the introduction of an orifice (a neck). The flow-focusing method can produce spherical microfluidic droplets with a diameter much smaller than the channel width.

The use of flow-focusing microfluidic devices for two-phase biocatalysis has been very limited despite the many advantages this method provides, such as controlled generation of spherical microdroplets (as opposed to ellipsoidal, see Figure 2), that float in the center of the channel, away from the walls, enabling mass transfer across the droplet interface. The only example of biocatalysis in flow focusing systems was published by Novak et al., who used this method for the synthesis of isoamyl acetate enzymes in microdroplets and the recovery of ionic liquids [45]. The use of microfluidic droplets enabled a significantly higher productivity of isoamyl acetate compared to the respective batch process. More details about this work can be found in Section 4.

**Figure 5:**
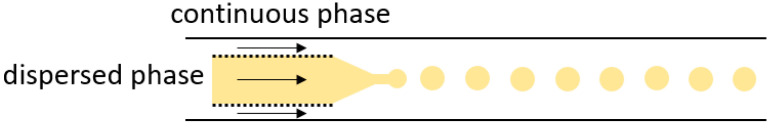
Droplet generation in a co-flowing microfluidic system. Small spherical droplets are formed in the channel resulting in droplet flow.

**Co-flowing**: Co-flowing is a method in which a capillary is embedded in a microchannel, and the dispersed and continuous phases flow through the capillary and the microchannel, respectively [46]. Unlike flow-focusing systems, co-flowing channels do not have a “neck-like” structure. The continuous phase does not squeeze the dispersed phase, but it is itself squeezed around the dispersed phase (Figure 5). This causes a destabilization at the dispersed phase front, which causes it to break into microdroplets. The size of droplets generated in co-flowing devices is controlled by the channel widths, the diameter of the capillary, and the flow rates used in experiments. In general, the co-flowing method produces well-controlled droplets with a diameter smaller than the width of the microfluidic channel. To date, no co-flowing system has been used in two-phase biocatalysis, presumably due to the complicated droplet generation principle and device fabrication process.

### 3.3. Droplet Generation and the Use of Surfactants

Droplet generation in microfluidic systems generally requires the use of surfactants, especially when droplets are generated using the flow-focusing or co-flowing methods. Surfactants are amphiphilic molecules commonly added to the continuous phase at small concentrations to promote the formation of new interfaces [47]. These molecules are adsorbed at the liquid–liquid interface to promote droplet stabilization and prevent coalescence [48]. Therefore, the addition of surfactants is important for stable droplet generation and manipulation and can also affect droplet properties such as their size.

However, as these amphiphilic molecules line the interface between the aqueous and organic phase, i.e., the droplet perimeter, it is expected that they also play a key role in the control of molecular exchange across the interface. Indeed, the rate of solute transport across a droplet interface through a surfactant monolayer has been shown to depend on many parameters such as the solute and surfactant size and structure, the density of the monolayer, and the curvature of the interface [49]. As mass transport across the droplet interface is a critical step during biocatalysis, the choice of suitable surfactants is also critical for high conversion rates. Additionally, surfactants have been shown to affect the catalytic activity of enzymes [50], and in turn, enzymes themselves can potentially serve as “surfactants” that stabilize droplet formation, as reported in the examples of lipase-catalyzed isoamyl acetate synthesis discussed in more detail below [41,45].

Most examples on biphasic biocatalysis in microreactors have relied on the use of polysorbate 20 (also known commercially as Tween 20) [28,50] or simply on the amphiphilic properties of the enzyme catalyst itself [41,51]. Previous work on enzyme evolution in microdroplets has primarily relied on fluorinated polyethers as surfactants [52,53]. Apart from available synthetic surfactants, a broad range of biological surface-active agents (biosurfactants) are also known today, ranging from various glycolipids or lipopeptides to surface-active proteins (e.g., hydrophobins) [54,55,56]. Biosurfactants have found various applications in industry due to their high interface activity [57]; their use in microfluidic droplet stabilization remains however limited.

### 3.4. Microfluidic Device Materials

When microfluidics are intended to host biotransformations, the choice of device material is critical. Aspects such as surface tolerance to solvents, channel hydrophobicity and gas permeability need to be carefully considered. The materials most commonly used in the fabrication of microreactors are glass, polycarbonate, and polytetrafluoroethylene (PTFE). Glass has the advantage of good transparency, chemical stability, and good biocompatibility. However, the fabrication of microchannels in glass requires expertise in microtechnology, as advanced processing using photolithography or laser microstructuring are necessary. Low entry manufacturing technologies such as 3D printing have also been investigated as they can be easily adopted by non-experts and used to create inexpensive devices [58]. However, the resolution that can be achieved using 3D printing methods such as stereolithography is limited (feature size in the order of a few hundred micrometers), and therefore, larger devices are produced, which requires a higher consumption of sample and reagents. Additionally, synthetic resins often display low tolerance against organic solvents. Polycarbonate offers the advantages of low cost, low water absorption, and good processing performance. It is the material of choice for microfluidics used in biomedical research and biological analysis, but its tendency to absorb some organic solvents makes it difficult to use for two-phase biocatalysis applications. However, surface coatings offer a solution to this problem. Mohr et al. used polycarbonate-based microfluidics coated with a 25-µm film of fluorinated ethylene propylene to create a recirculating, two-phase flow microbioreactor. The fluoropolymer coating was used to increase the chemical resistance and hydrophobicity of the microchannels [59]. Finally, PTFE offers inertness to chemicals and solvents, antifouling properties, moderate gas permeability, and low non-specific protein adsorption compared to polycarbonate. It is therefore widely used in the field of biphasic biocatalysis, especially used in microreactors made from microcapillary tubing [60].

## 4. Examples of Two-Phase Biocatalysis in Microdroplets

**Figure 6:**
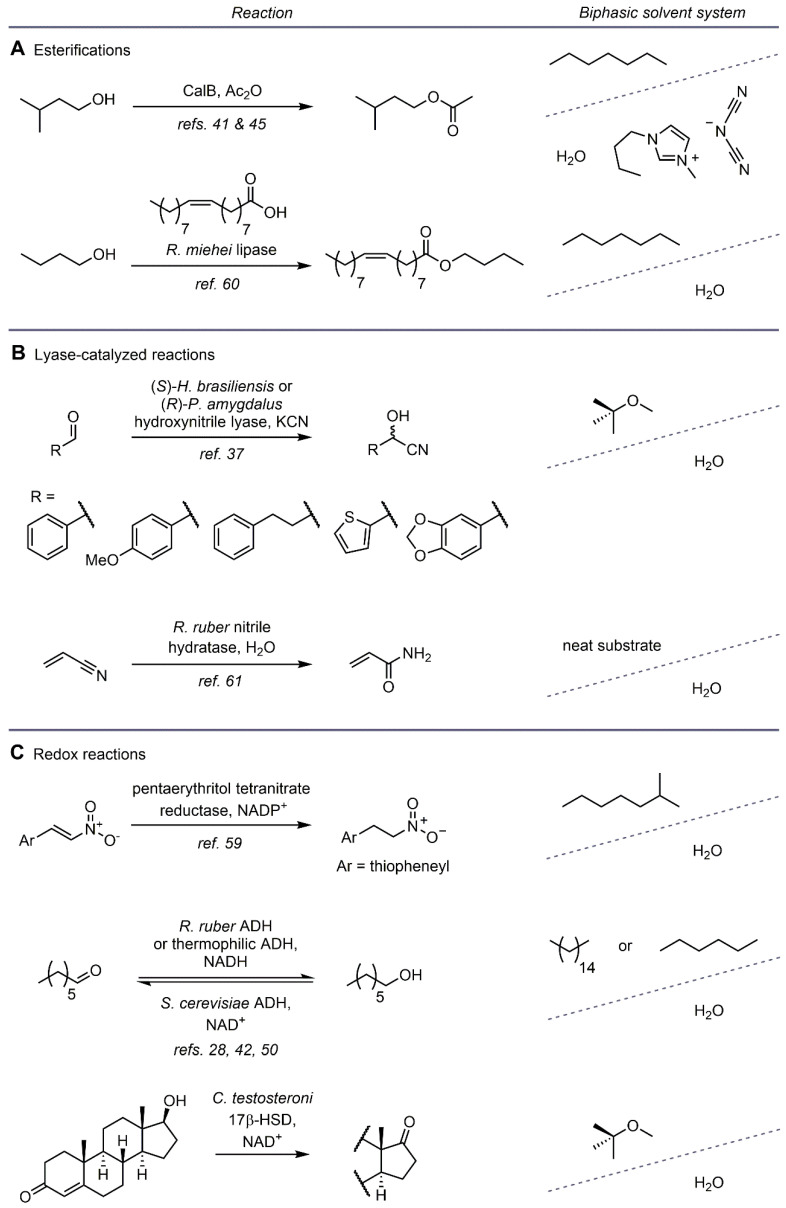
Biocatalytic two-phase reaction examples performed in microfluidic droplets.

Biocatalytic esterifications, redox reactions, and lyase-catalyzed conversions making use of two liquid phases of different polarity have been performed in microdroplets (see Figure 6). These biphasic biotransformations have been used to increase substrate concentration in the reaction, efficiently extract product into the non-aqueous phase for simplified downstream processing, or reduce substrate and/or product inhibition of the enzyme. In the following paragraphs, notable examples of two-phase biocatalysis in microdroplets are discussed. These examples include work performed in both microfabricated reactors, as well as droplets generated in similar device architectures using microcapillaries (capillary-based microreactors).

In 2009, Znidarsic–Plazl and coworkers reported the CalB-catalyzed synthesis of isoamyl acetate, a flavor, and fragrance compound used in food, cosmetics, and pharmaceutical industry, from isoamyl alcohol and acetic anhydride in a continuously-operated Ψ-shaped droplet microreactor made out of glass [41]. The employed two-phase system consisted of a water-miscible ionic liquid and *n*-heptane. While all reagents and the aqueous enzyme solution were suspended in the ionic liquid phase, the product (isoamyl acetate) was efficiently extracted into the hydrophobic organic phase. Using a 3:1 ratio of alcohol to acetic anhydride and a microfluidic flow pattern that resulted in a combination of long slugs (ellipsoidal droplets) and circulating fine droplets of *n*-heptane in the continuous ionic liquid phase, enabled an almost 3-fold productivity increase compared to the respective batch process. This significant improvement was mainly attributed to more efficient mixing and hence a much higher interfacial area between liquids within the microchannel. When a flow-focusing microfluidic chip was used for the same enzymatic reaction, uniform *n*-heptane droplets were formed in the ionic liquid phase [45]. This resulted in an even higher interfacial area, across which the reaction and product extraction takes place, with the amphiphilic lipase positioned at the ionic liquid-*n*-heptane interface. Moreover, the integration of a membrane-based phase separator allowed the reuse of the enzyme-containing ionic liquid over several cycles.

In another approach, the performance of capillary microreactors with various inner diameters was compared, when *Rhizomucor miehei* lipase was used for the esterification of oleic acid with 1-butanol for biodiesel production [60]. The aqueous phase containing the dissolved enzyme and the organic phase comprising fatty acids and butanol in *n*-heptane were introduced through a Y-junction resulting in a slug-flow profile. Two microreactor materials were used: hydrophobic PTFE or hydrophilic stainless steel. In PTFE channels, aqueous droplets were formed within the organic slug. In stainless steel channels, droplets of the organic phase were formed within the continuous aqueous phase. An increase in the volumetric organic-to-aqueous phase flow ratio significantly improved enzyme performance in the stainless steel microreactor in contrast to the PTFE-based one. This difference was explained by a larger increase in the interfacial area when the organic phase is moving through the continuous aqueous phase in the form of long droplets, as compared to the short aqueous droplets in a continuous organic phase observed in the PTFE-based microreactor [60].

Next to those biocatalytic esterifications, Koch et al. reported the successful formation of C-C bonds using crude lysates of two different hydroxynitrile lyases in specially designed microreactors [37]. Enzyme-catalyzed addition of HCN to different aldehydes using a two-liquid phase system under undefined slug flow in Y-shape microfluidics yielded α-cyanohydrins with excellent enantioselectivity. The organic phase made of MTBE contained the respective aldehyde, while the cyanide (in form of KCN) was provided via the enzyme-containing aqueous phase. The authors further demonstrated that this setup could be applied successfully for the screening of different reaction parameters using only minute amounts of reagents and a significantly shorter time compared to batch experiments. As another example, the industrially relevant hydration of acrylonitrile by nitrile hydratase to form acrylamide was reported using a membrane dispersion, stainless steel microreactor [61]. In membrane dispersion microreactors, the two phases are separated by a membrane. Droplets are formed when one phase is pushed into the other through the membrane. In the work discussed here, acrylonitrile was added as a substrate to the continuous aqueous phase, which contained whole *Rhodococcus ruber* TH3 cells as the catalyst. Membrane dispersion was used to produce acrylonitrile droplets with 25–35 µm diameter. The large surface area of these microdroplets significantly enhanced mass transfer and led to high acrylamide concentration (45.8 wt%) within 35 min of reaction.

Selected biocatalytic redox reactions in microfluidic droplets have also been reported, which utilize key advantages of microdroplets and have served as model systems for process optimization. Mohr and colleagues examined the performance of a promiscuous pentaerythritol tetranitrate reductase under anaerobic conditions in a recirculating continuous-flow microbioreactor [59]. Coating the polycarbonate bioreactor walls with thick hydrophobic fluoropolymer enabled the formation of stable aqueous droplets in isooctane using a T-junction. Installation of spectroscopic cells in the recirculating organic and aqueous channels allowed UV-spectroscopic reaction monitoring based on the absorbance of the substrate/product and the NADPH cofactor. By implementing a NADPH recycling system in the aqueous phase, the authors also investigated the performance of the bioreactor when it comes to the reduction of selected olefins. They found an improved performance compared to analogous reactions in batch, presumably due to a combination of increased mass transfer granted by a higher surface-to-volume ratio, as well as efficient product removal from the aqueous phase. To further study mass transfer limitations in biphasic redox reactions, Buehler and coworkers examined the effect of various process variables on the reduction of 1-heptaldehyde by a thermophilic alcohol dehydrogenase [28]. An aqueous buffer containing the enzyme, the cofactor, and recycling system based on formate dehydrogenation was mixed with a hexadecane phase at a T-junction and formed slugs in the organic phase. The authors probed the effects of flow rate, capillary diameter, phase ratio, as well as enzyme and substrate concentrations under segmented flow conditions. By optimizing these parameters, the system could be tuned for efficient enzyme usage, while maintaining high productivity. Remarkably, despite the enzyme’s inhibition at >1 mM substrate in the aqueous phase, the mass transfer optimization performed in this capillary system enabled the efficient feeding of substrate and concurrent product removal, achieving product titers of almost 40 mM. In an additional study, the same authors utilized slugs formed in a capillary T-junction to address the low stability of the dehydrogenase under operational conditions [50]. Inactivation of the enzyme at the liquid interface was successfully eliminated through the application of low concentrations of Tween 20 as a surfactant, most likely via stabilization of the phase barrier. Finally, Zelic and colleagues used the reverse reaction as a model system for the evaluation of kinetic parameters in a continuously operated Y-shaped tubular microreactor [42]. *Using Saccharomyces cerevisiae* alcohol dehydrogenase, the authors examined the oxidation of hexanol in a biphasic system composed of buffer and hexane. Compared to batch experiments, the authors found a drastic increase in the reaction rate as well as a complete elimination of product inhibition.

A further example comes from our own recent work, where we used flow-focusing microfluidic devices to perform testosterone dehydrogenation catalyzed by the 17β-hydroxysteroid dehydrogenase (17β-HSD) from *Comamonas testosteroni* [62] in microfluidic droplets. Microfluidic devices were fabricated in glass using femtosecond laser ablation. The buffer solution containing the enzyme was used as the aqueous carrier phase and methyl tert-butyl ether (MTBE) containing testosterone was used as the organic phase confined in the microdroplets. For reactions performed in microfluidics with 10 mM steroid concentration, a 91% conversion of testosterone to androstenedione was obtained in only 40 s of reaction time, resulting in a space-time yield (STY) of 234 g L^−1^ h^−1^. In comparison, respective batch reactions yielded 85% conversion after 10 min reaction time, resulting in a STY of only 14.6 g L^−1^ h^−1^.

The works discussed above underscore the value of microdroplets in the testing and optimization of biphasic biocatalytic processes that typically suffer from product inhibition and low mass transfer rates. At the same time, many questions arise about the utility of droplet microreactors and the root of the underutilization of such systems in biocatalytic process design.

## 5. Conclusions and Future Perspective

As presented in detail above, microfluidic systems offer a unique set of advantages that make them particularly suitable as reaction vessels for two-phase biocatalysis. These advantages include continuously operated reactions, short diffusion paths, and tremendously increased mass transfer rates. Biphasic biocatalytic processes have been reported in microfluidic devices, primarily ones utilizing laminar flow configurations [38,39]. Despite the additional advantages offered by droplet-based microfluidics, such as dramatically increased surface-to-volume ratios, their use in two-phase biocatalysis remains limited. This is mainly due to the complexity associated with microfluidic device fabrication and the establishment of a stable droplet flow. Commercial droplet generation and control systems (such as the Elveflow Microfluidic Droplet Pack) offer an out-of-the-box solution to this problem as their use requires no expertise in microtechnology. The few examples of two-phase biocatalysis in microdroplets discussed above primarily utilize simple T-, Y-, or Ψ-junctions to establish slugs [37,41,50,59,60]. Slug flow can be realized at lower flow rates compared to droplet flow and does not typically require the use of surfactants or channel surface treatment. Additionally, slug flow can be established in microcapillary tubes, making this method more accessible to researchers with no access to microfabrication facilities or specialized equipment. However, slugs usually have large, variable diameters that are difficult to control. This means lower surface-area-to-volume ratio compared to microfluidic droplets generated in flow-focusing systems and less controlled experiments. Flow-focusing devices have a relatively complicated architecture and droplet generation often requires device and process optimization. However, the droplets produced in these systems have a uniform, defined spherical shape and a diameter that is smaller than the channel size. This ensures a large interfacial area between the two phases in biphasic biocatalysis, which in turn should lead to enhanced conversion and reaction rates because of increased mass transport across the interface. Stable microdroplets that do not coalesce usually require the use of a surfactant. Surfactants line the interface between the aqueous and organic phase, and it is thus expected that they would also control molecular exchange across the interface. On the other hand, as enzymes can be inactivated when in contact with organic solvents, the addition of surfactants could have a positive effect, by eliminating enzyme inactivation at the liquid-liquid interface as shown in one literature example [50]. Similarly, a large interfacial area between the aqueous and organic phases in microdroplet systems might introduce an additional risk for enzyme inactivation, as enzyme inactivation in liquid−liquid bubble column systems was found to be proportional to the interfacial area [63]. A large interfacial area is, however, a requirement for enhanced mass transfer. The tradeoff between maximized mass transfer and high enzyme inactivation rates in small droplets with large surface-to-volume ratios remains to be investigated, as it was never explicitly studied in droplet microfluidics. It should be noted here, however, that such enzyme inactivation will largely depend on the solvent system used as well as the employed enzyme and it hence needs to be addressed on a case-by-case basis.

To directly compare and evaluate the effectiveness of each method when employed in two-phase biocatalysis is a difficult task. Although individual studies often compare results obtained in batch process to results obtained in droplet-based processes, a comparison between two-phase biocatalysis in batch, laminar flow, slug flow, and droplet flow has not been performed. Nevertheless, comparisons between batch processes and droplet processes consistently show higher conversion in droplets, explained by enhanced mass transport, product removal (lower product inhibition), and reaction rates.

As evident by the literature review and discussion in this manuscript, microfluidic droplets have the potential to improve biphasic biocatalysis but have so far been grossly underutilized. Biocatalytic reactions that are product inhibited could particularly benefit from fast mass transfer that allows in situ product extraction into microdroplets. Similarly, reactions that are substrate inhibited benefit from chemical compartmentalization and short diffusion paths. Although slow biocatalytic reactions might not be compatible with conventional continuous-flow microfluidics due to the short liquid residence time in these devices (seconds to few minutes), longer channels, stationary reaction chambers, or the use of long capillaries offer alternatives that can make long reactions in microdroplets feasible.

In summary, several questions related to the application of this technology in biocatalysis remain unanswered, including:

- Which method of droplet generation produces the best results and why?
- Which type of flow (laminar, slug, droplet) can deliver the best results and why?
- What are the effects of surfactants on biotransformation?
- Is there a tradeoff between enzyme inactivation and enhanced mass transfer when the interfacial area increases?

Answers to these questions would provide researchers with the tool kits necessary to make informed decisions about the most suitable technology to use for their reactions. This would lower the barrier for the adaptation of droplet microfluidics in biocatalysis laboratories and would fuel development in this direction.
